# Systematic evaluation for the causal effects of blood metabolites on osteoporosis: Genetic risk score and Mendelian randomization

**DOI:** 10.3389/fpubh.2022.905178

**Published:** 2022-08-25

**Authors:** Xing-Hao Yu, Rong-Rong Cao, Yi-Qun Yang, Lei Zhang, Shu-Feng Lei, Fei-Yan Deng

**Affiliations:** ^1^Center for Genetic Epidemiology and Genomics, School of Public Health, Medical College of Soochow University, Suzhou, China; ^2^Jiangsu Key Laboratory of Preventive and Translational Medicine for Geriatric Diseases, Soochow University, Suzhou, China

**Keywords:** metabolite, osteoporosis, bone mineral density, Mendelian randomization, genetic risk score

## Abstract

**Purpose:**

Osteoporosis is associated with metabolic alterations, but the causal roles of serum metabolites on osteoporosis have not been identified.

**Methods:**

Based on the large individual-level datasets from UK Biobank as well as GWAS summary datasets, we first constructed genetic risk scores (GRSs) for 308 of 486 human serum metabolites and evaluated the effect of each GRS on 2 major osteoporosis phenotypes, i.e., estimated bone miner density (eBMD) and fracture, respectively. Then, two-sample Mendelian Randomization (MR) was performed to validate the casual metabolites on osteoporosis. Multivariable MR analysis tested whether the effects of metabolites on osteoporosis are independent of possible confounders. Finally, we conducted metabolic pathway analysis for the metabolites involved in bone metabolism.

**Results:**

We identified causal effects of 18 metabolites on eBMD and 1 metabolite on fracture with the GRS method after adjusting for multiple tests. Then, 9 of them were further validated with MR as replication, where comprehensive sensitive analyses proved robust of the causal associations. Although not identified in GRS, 3 metabolites were associated with at least three osteoporosis traits in MR results. Multivariable MR analysis determined the independent causal effect of several metabolites on osteoporosis. Besides, 23 bone metabolic pathways were detected, such as valine, leucine, isoleucine biosynthesis (*p* = 0.053), and Aminoacyl-tRNA biosynthesis (*p* = 0.076), and D-glutamine and D-glutamate metabolism (*p* = 0.004).

**Conclusions:**

The systematic causal analyses strongly suggested that blood metabolites have causal effects on osteoporosis risk.

## Introduction

As a systemic metabolic bone disease, osteoporosis is widespread and associated with increased fracture risk in post-menopausal women of advanced age. Main causes of osteoporosis include the defects of bone formation during the growth period, impaired bone formation due to decreased osteoblast differentiation, and several pathological processes leading to increased bone resorption (1). Increasing age, female, sex hormone deficiency, family history of osteoporosis, excessive alcohol consumption, smoking, and various chronic medical conditions are considered osteoporosis risk factors (2).

Metabolites, as mediators, play a particularly important role in bone metabolism. The metabolic state of the body is a major determinant of bone health. According to the study of the ovariectomized rat osteoporosis model, the altered metabolites were mainly related to the metabolism of energy, lipids, and amino acids (3). Although several metabolites were observed to be related to osteoporosis in population-based cohorts (4), there are still no systematical evaluations of the effects of metabolites on osteoporosis. In addition, traditional studies were hard to be used to identify and establish the potentially causal links between blood metabolites and osteoporosis due to the unavoidable confounders (5). In recent years, numerous studies have integrated metabolomics with high-throughput genotypes to estimate the effects of genetic variants on metabolic phenotypes *via* genome-wide association studies (GWASs), and identify thousands of genetic loci associated with metabolic phenotypes (6). Based on large GWAS datasets, recent genetic risk scores (GRSs) and Mendelian randomization (MR) have been proved to be powerful in assessing the etiology of complex diseases, as unknown confounding factors could be effectively controlled (7). Although previous studies have found several metabolites causally linked to hip or spine bone miner density (BMD) (8, 9), the causal roles of metabolites in estimated BMD (eBMD) and osteoporotic fractures have not been evaluated.

**Figure 1:**
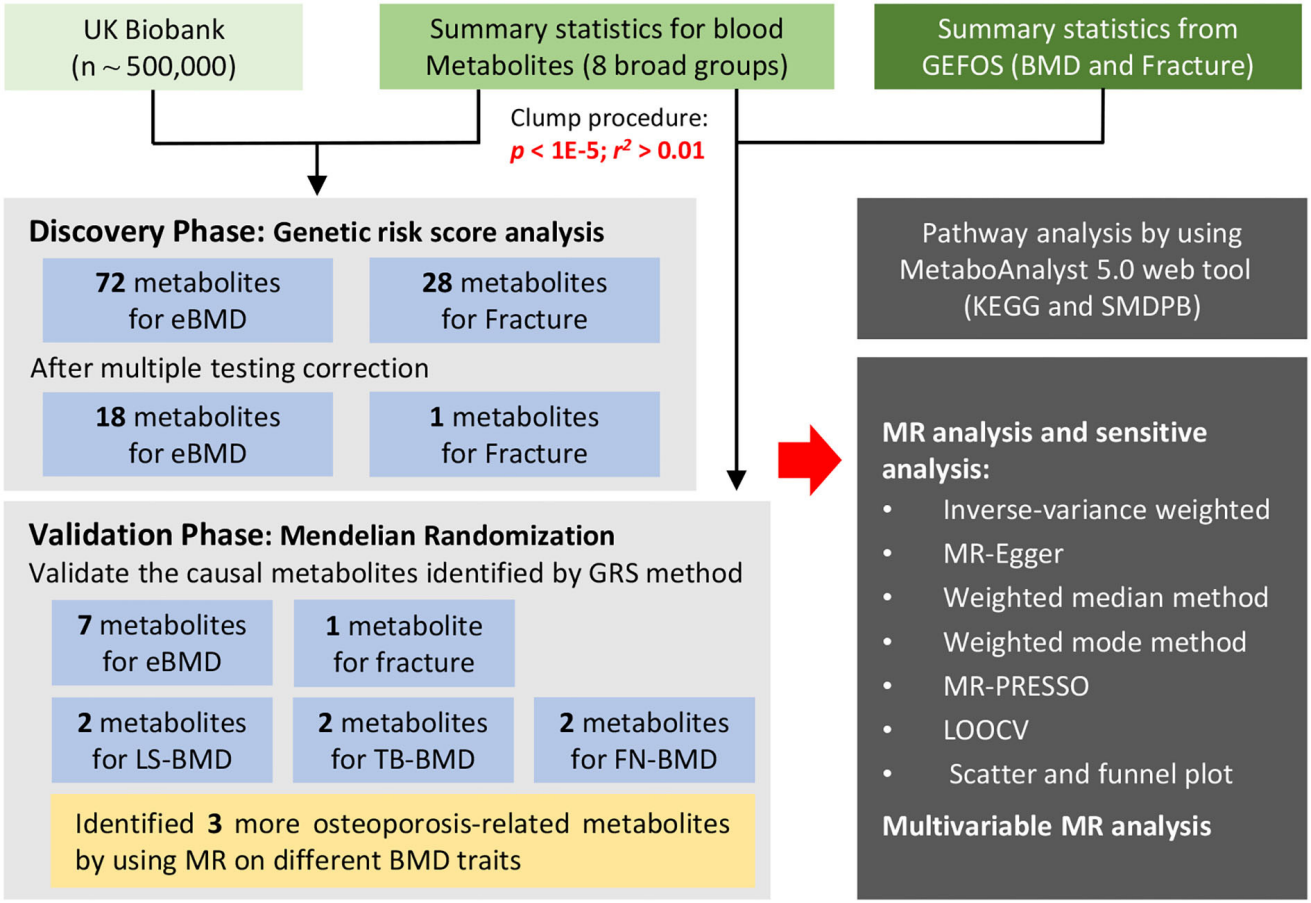
The overview of the research workflow.

The UK Biobank is a large GWAS dataset in the world (~500,000 individuals), which offers unprecedented opportunities to screen populations for clinical biomarkers based on the genotype data of the individuals (10). Based on UK Biobank, we systematically evaluated the causal associations between blood metabolites and osteoporosis in conjunction with large-scale summary statistics from previous GWAS to identify common metabolic mechanisms between BMD and fracture. Specifically, we constructed GRS for each metabolite and assessed their association with eBMD and fracture separately. Furthermore, we conducted two-sample MR analyses to explore the causative metabolites of BMD and fracture. The biological functions of the identified causal metabolites were annotated by metabolic pathway analysis. Besides, independent causal metabolite on osteoporosis was verified by utilizing a novel multivariate MR method. An overview of our research workflow is presented in Figure 1.

## Materials and methods

### UK biobank datasets

The individual-level clinical and genetic datasets were accessed from the UK Biobank (Application 41542), a project that recruited ~500,000 individuals aged 40–69 years. (10). The individuals' medical records in UK Biobank were based on the International Classification of Diseases (ICD), Tenth Revision (ICD-10-CM), and Ninth Revision (ICD-9-CM). BMD-related variables, including fracture (Field ID: 6151), eBMD (Field ID: 3084, 3148, 4105), age (Field ID: 21003), gender (Field ID: 31), height (Field ID: 50), and weight (Field ID: 21002) and the top 10 genetic principal components, were extracted for analyses. For individuals, we have retained individuals of British ancestry and with genotype data. For SNPs, we performed quality control, as described previously (11). The genotyping process and quality control process used in the UKB study have been described elsewhere (10). Specifically, we restricted our analysis to high-quality Haplotype Reference Consortium-imputed autosomal variants. We retained SNPs with MAF >0.02; imputation information score >0.5; missing ratio >0.95; and Hardy-Weinberg, *p* > 1 × 10^−7^. For sample quality control, we excluded individuals with unsuccessful genotyping, non-white British, and at least one kinship determined. Finally, our final genetic analysis included up to ~330,000 individuals of independent European ancestry and ~9,000,000 high-quality single nucleotide polymorphisms (SNPs).

### Metabolome-wide GWAS data sources

We downloaded summary association statistics from the most comprehensive genetic study on human metabolism, which was publicly available on the Metabolomics GWAS Server (website: http://metabolomics.helmholtz-muenchen.de/gwas/) (6). Based on 7,824 individuals from 2 European population cohorts, a total of 486 metabolites were measured by the MS (Metabolon) platform, and GWAS analyses were performed in the HapMap2-based-imputed genotype dataset. After excluding 177 unknown metabolites, 309 known metabolites were adopted in the present study. The known metabolites can be classified into 60 subclasses and 8 broad classes according to the Kyoto Encyclopedia of Genes and Genomes (KEGG) pathways. For each metabolite, an average of 2.1 million SNPs were reserved with GWAS summary statistics. In order to ensure the rationality of our analysis, we carried out quality control of SNPs, specifically including removing non-biallelic SNPs, all SNPs with strand-ambiguous alleles, SNPs without rs IDs, duplicated rs IDs or base pair position, SNPs not in 1,000 Genomes Project Phase 3, SNPs whose base pair positions or alleles do not match those in 1,000 GP Phase 3, SNPs with imputation INFO <0.9, and all SNPs on chromosome X, Y (12).

### GWAS data sources for osteoporosis fracture and BMD

The summary association statistics of fracture and eBMD, including genotyping and imputed data from up to ~500,000 European participants, were publicly available from the Genetic Factors for Osteoporosis (GEFOS) Consortium (http://www.gefos.org/) (13). After quality control, 13,977,204 SNPs measured for 426,795 (53,184 fracture cases) individuals and 13,681,377 SNPs measured for 426,824 individuals with eBMD data were obtained. The fracture was identified by a questionnaire, and the data were extracted with the ICD-10-CM codes. Additionally, life-course total body (TB)-BMD and DXA-BMD at multiple skeletal sites [lumbar spine (LS)-BMD, forearm (FA)-BMD, femoral neck (FN)-BMD] were extracted to further explore the associations with metabolites. GWAS meta-analysis for TB-BMD involved 30 epidemiological studies and included 66,628 individuals. Genotype imputation was performed using the 1,000 Genomes Phase 1 v.3 (March 2012) reference panel for each study, and GWAS analysis contained ~30,000,000 SNPs. Association analysis was performed after adjusting for age, weight, height, and genomic principal components, as well as any other covariates (e.g., recruitment center) (14). For DXA-BMD, FN-BMD contained 32,735 individuals, LS-BMD contained 28,498 individuals, and FA-BMD contained 8,143 individuals. All BMD data were normalized to mean zero and standard deviation 1. Then, the association analyses were conducted on approximately ~9,000,000 SNPs, adjusted for sex, age, age squared, and weight (15). Information of the above summary data is summarized and presented in Supplementary Table S1.

### Selection of instrumental variables (IVs)

According to the directions (16, 17), IVs for each metabolite were selected with the clumping procedure at a loose threshold in PLINK software (version v1.90 b3.38) (18). Specifically, when clumping, we set the significance threshold at 1.00E-5, the linkage disequilibrium *r*^2^ at 0.1, and used the 500 KB window from 1,000 Genomes Projects as a reference panel. One of the 309 known metabolites did not have any significant locus. A total of 3 to 631 independent genetic variants were selected as IVs for each of the 308 metabolites. The SNP information for five identified causal metabolites is also presented in Supplementary Table S2. Besides, we also selected IVs of BMD for further reverse-directional MR analysis. The same threshold of LD *r*^2^ and windows as for metabolites was used, with the exception that the significance threshold was set at 5E-8. Finally, 307, 14, 23, 25, 105, and 4 SNPs were retained for eBMD, fracture, FN-BMD, LS-BMD, TB-BMD, FA-BMD, specifically. To quantitatively verify whether the selected SNPs were strong instruments, we calculated the proportion of phenotypic variation explained (PVE) and the F statistic of instruments for each metabolite.

### GRS analysis in UK biobank

To assess the potentially causal role of metabolites in osteoporosis, the GRS for each metabolite can be calculated by


(1) $$\begin{array}{c} {\text{GRS}}_{m}\;=\;\sum_{i\;=\;1}^{I}X_{i}{\hat{\beta }}_{i} \end{array}$$

where ${\hat{\beta }}_{i}$ is the estimated effect for *i*^th^ IV associated with metabolite *m*, and *X*_*i*_ is the individual-level genotypes of the index SNP in the UK Biobank. Then, multivariable linear regression was utilized to estimate the effect size of each metabolite on eBMD with the standardized GRS after adjusting for covariates of age, sex, weight, height, and the tops 10 principal components. Significant causal metabolites of osteoporosis were determined by using Bonferroni correction with *p-*values <1.03E-04 (0.05/486) for eBMD and fracture. The suggestive metabolites were identified at a nominal significance level (*p* < 0.05).

### Metabolic pathway analysis

To explore the functions of identified metabolites, MetaboAnalyst 4.0 (https://www.metaboanalyst.ca/) was used to conduct metabolic pathway analysis (19). To obtain comprehensive and credible pathway analysis results, all significant metabolites related to eBMD/fracture or four BMD traits, respectively (*p* < 0.05), were used. Herein, two libraries, including the Small Molecule Pathway database (SMPDB) and the Kyoto Encyclopedia of Genes and Genomes (KEGG) database, were utilized, and the significance level of pathway analysis was set at 0.10.

### Estimation of causal effect and sensitivity analyses

Inverse variance-weighted (IVW) analysis was mainly performed to evaluate the causal effect of potentially causal metabolites on osteoporosis. Cochran's Q statistic and associated *p*-values were calculated to examine the heterogeneity and pleiotropy effect. If the null hypothesis is rejected, which indicates that one or more variants may be multivariate, random-effect IVW is performed instead of fixed-effect IVW (20). Additionally, to control for the horizontal pleiotropy, three complementary sensitive analyses were conducted: (1) the weighted median-based method, which gave a consistent estimate when half of the weight was from valid IVs (21); (2) The mode-based method, which provides a consistent effect estimate if most of the genetic variants are valid instruments as the sample size increases (22); (3) MR-Egger regression, which provides a powerful method to test the horizontal pleiotropy (23). LOO analysis and the MR pleiotropy residual sum and outlier (MR-PRESSO) method were utilized to validate possible horizontal pleiotropic outliers that might affect the estimation substantially (24). Finally, to ensure the robustness of our results, we performed MR analysis based on IVs selected under different thresholds (i.e., *P*-values and *r*^2^). To rule out the possibly bi-directional association between metabolites and BMD, we also conducted reverse MR analysis regarding four BMD traits and identified metabolites, respectively.

### Multivariable MR analysis

We utilized novel multivariable MR analysis to estimate the independent causal effect of certain metabolites on BMD after controlling for other significant metabolites due to the possible horizontal pleiotropy (25). SNPs associated with these metabolites were used as IVs, and their corresponding information, as well as that of BMD, was eventually incorporated into our MR framework:


(2) $$\begin{array}{l} \begin{array}{c} {\hat{\lambda }}^{BMD}={\hat{\lambda }}^{metabolite_{1}}\beta_{1}+\cdots +{\hat{\lambda }}^{metabolite_{j}}\beta_{j} \end{array}\\ \begin{array}{c} +\varepsilon ,\;\varepsilon^{\tilde{\;}}\;N(0,\;\sigma^{2}) \end{array} \end{array}$$

Here, $\hat{\lambda }$ is the marginal effect size of instruments, σ^2^ represents the variance for residual term ε, β_1_ and β_*j*_ represent the causal effect of the first and second metabolites on BMD, respectively. Then, we estimated the effect size of β_1_ and β_*j*_ with the weighted least squares method.

All statistical analysis was performed with the R 3.5.1 software. An MR analysis was conducted with the ‘MendelianRandomization' package (version 0.4.3) (26), and MR-PRESSO was performed with the MR-PRESSO package (24).

## Result

### Identification of eBMD-associated metabolites by using GRS analysis

**Figure 2:**
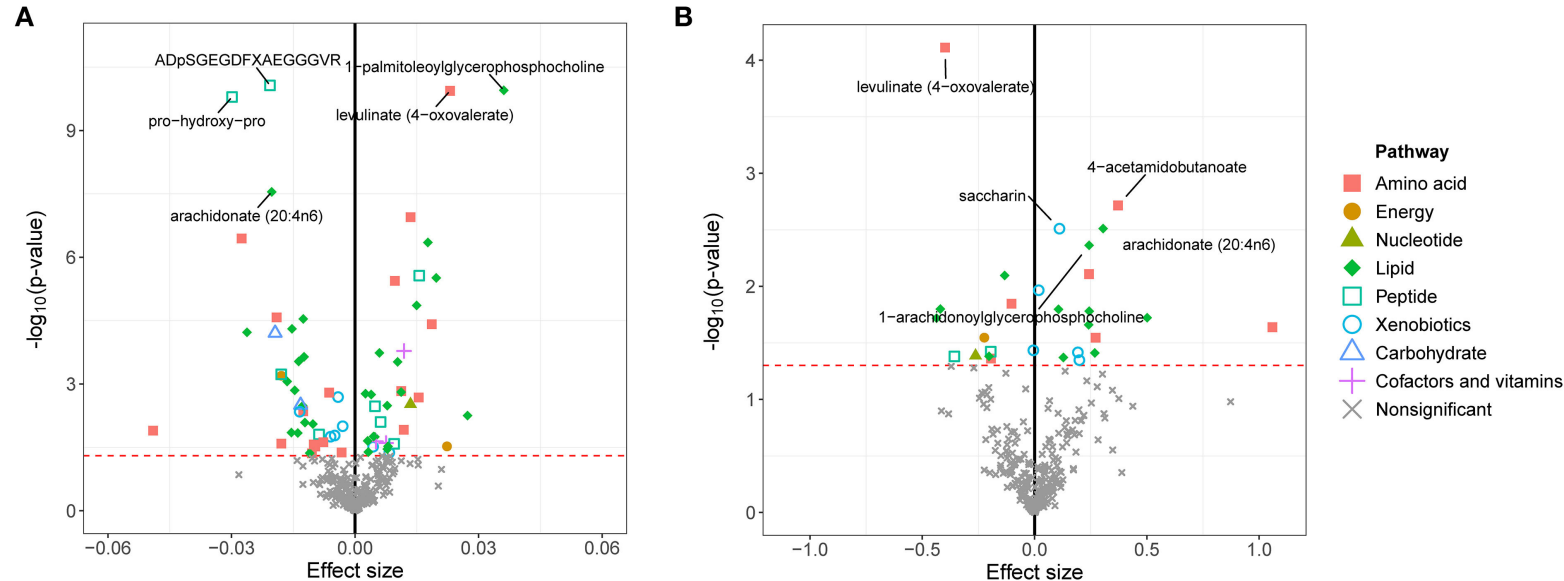
GRS associations between serum metabolites and eBMD **(A)** or fracture **(B)**. The x-axis represents the effect size, and the y-axis represents the –log_10_(*p*). Five top metabolites with minimal q-values were annotated. Significantly associated metabolites (*p* < 0.05) were marked with corresponding pathway information. eBMD, estimated bone mass density.

We first calculated the GRSs for 308 known metabolites from the large-scale UK Biobank database and found significant associations between these GRSs and eBMD/fracture by multivariable linear regression analysis after adjusting for covariates (Figure 2). Suggestive metabolites associated with osteoporosis, including 72 for eBMD and 28 for fracture, were identified (Supplementary Table S3). After Bonferroni correction, a total of 18 known metabolites were significant for eBMD (*p* <1.03E-04) (Figure 2A). Among the 18 metabolites, 8 belong to the lipid pathway, 6 belong to the amino acid pathway, 3 belong to the peptide pathway, and 1 belongs to the carbohydrate pathway. For fracture, only one significant metabolite, i.e., levulinate (4-oxovalerate) from the amino acid pathway, was identified after Bonferroni correction (Figure 2B).

### Metabolic pathway analysis

Metabolic pathway analysis on suggestive GRS-identified metabolites (72 for eBMD and 28 for fracture) showed that the eBMD-associated metabolites were enriched in 4 pathways (*p* < 0.10), including “Caffeine metabolism” (*p* = 0.010), “valine, leucine, isoleucine biosynthesis” (*p* = 0.053), “Aminoacyl-tRNA biosynthesis” (*p* = 0.076) (Supplementary Figure S1A, KEEG pathways) and “Alpha Linolenic Acid and Linoleic Acid Metabolism” (*p* = 0.041, Supplementary Figure S1B, the SMPDB pathway). The fracture-associated metabolites were enriched in “Alpha Linolenic Acid and Linoleic Acid Metabolism” (*p* = 0.008) and “Methyl histidine Metabolism” pathways (*p* = 0.087) (Supplementary Figure S2).

Additionally, metabolites identified with the IVW method for other traits were found to be enriched in seven metabolic pathways (Supplementary Table S4).

### Validation of causal effect of metabolites on fracture and BMD with MR analysis

**Figure 3:**
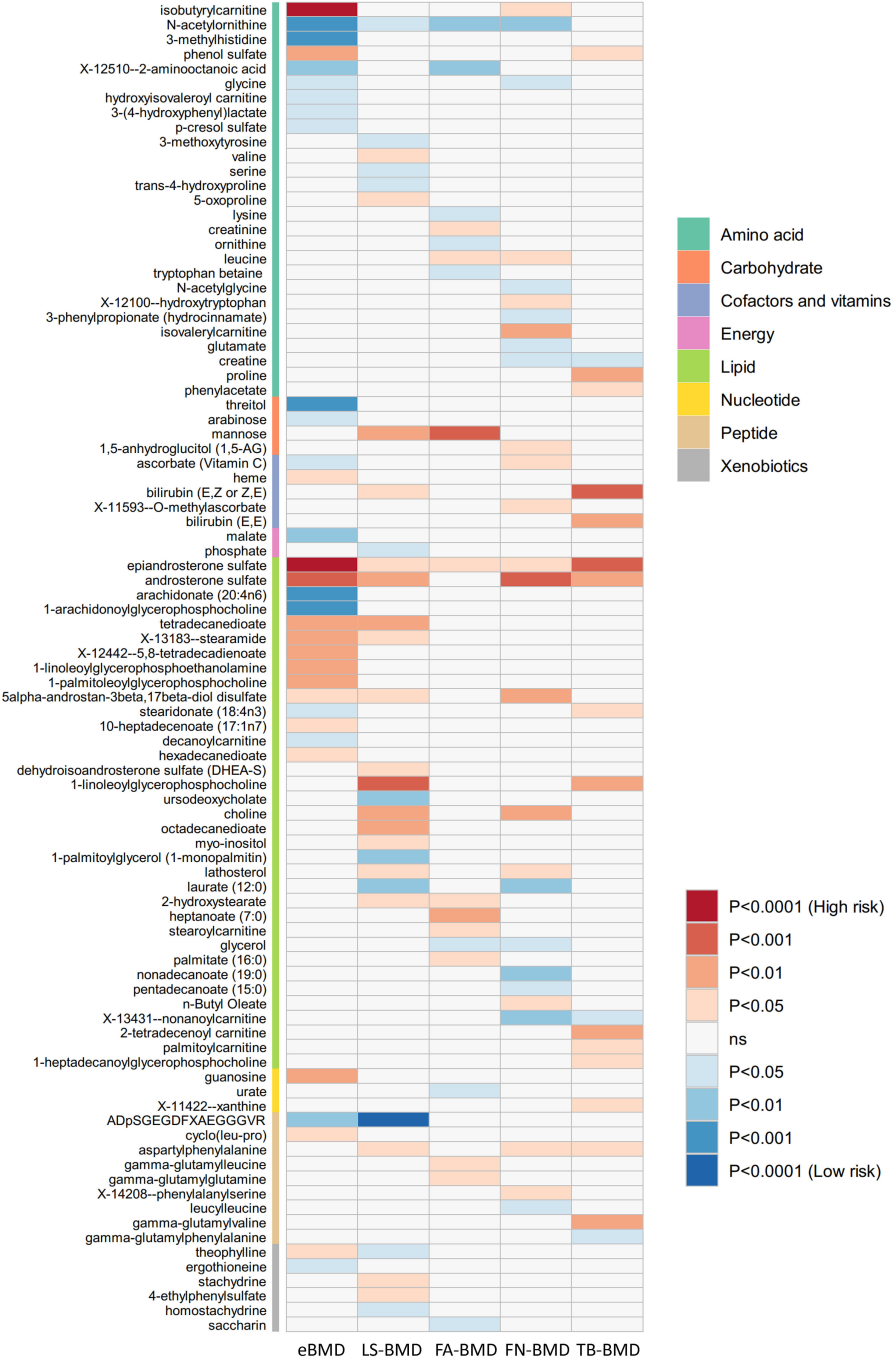
Mendelian randomization associations of serum metabolites on five BMD phenotypes that derived from the IVW analysis. IVW, inverse-variance weighted; eBMD, estimated bone mineral density; TB-BMD, total body bone mineral density; LS-BMD, lumbar spine bone mineral density; FA-BMD, forearm bone mineral density; FN-BMD, femoral neck bone mineral density.

**Figure 4:**
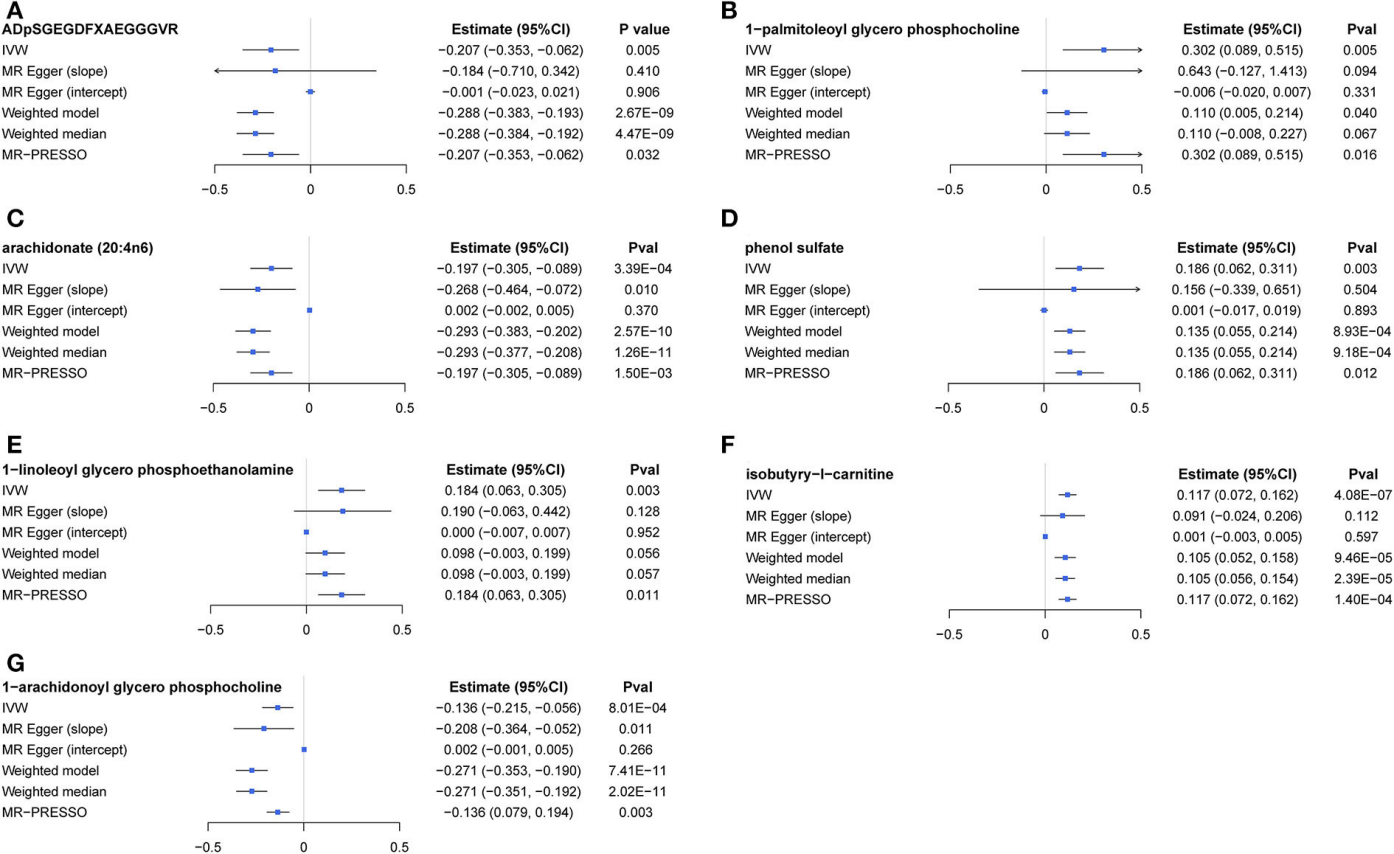
A forest plot about associations between 7 causal metabolites and eBMD. **(A)** *ADpSGEGDFXAEGGGVR*; **(B)** *1-palmitoleoyl glycerol phosphocholine*; **(C)** arachidonate (20:4n6); **(D)** phenol sulfate; **(E)** 1-linoleoyl glycerophosphoethanolamine; **(F)** isobutyryl-l-carnitine; **(G)** 1-arachidonoyl glycerol phosphocholine. IVW, inverse-variance weighted.

The causal relationship between GRS-identified metabolites and osteoporosis was further estimated by the two-sample MR method for eBMD/fracture (Supplementary Table S3) and DXA-BMD (Supplementary Table S5), respectively. The metabolites' association signal distribution among various sites of BMD and fracture is presented in Figure 3. For eBMD, 7 metabolites were significant (*p* < 0.05) by using the IVW method (Table 1), including 4 metabolites from the lipid pathway, 2 from the amino acid pathway, and 1 from the peptide pathway. Specifically, positively causal effects on eBMD were identified for four metabolites, including isobutyryl-l-carnitine (β_IVW_ = 0.117, *P*_IVW_ = 4.08E-07), 1-palmitoleoylglycerophosphocholine (β_IVW_ = 0.302, *P*_IVW_ = 0.005), phenol sulfate (β_IVW_ = 0.186, *P*_IVW_ = 0.003), and 1-linoleoylglycerophosphoethanolamine (β_IVW_ = 0.184, *P*_IVW_ = 0.003). Negatively causal effects were identified for three metabolites, including ADpSGEGDFXAEGGGVR (β_IVW_ = −0.207, *P*_IVW_ = 0.005), arachidonate (20:4n6) (AA) (β_IVW_ = −0.207, *P*_IVW_ = 0.005), and 1-arachidonoylglycerophosphocholine (β_IVW_ = −0.136, *P*_IVW_ = 8.01E-04). Of the above 7 eBMD-associated metabolites, 3 were associated with DXA-BMD as well. For example, ADpSGEGDFXAEGGGVR is negatively associated with LS-BMD (β_IVW_ = −0.369, *P*_IVW_ = 1.08E-05), phenol sulfate is positively associated with TB-BMD (β_IVW_ = 0.164, *P*_IVW_ = 0.012), and isobutyryl-l-carnitine is positively associated with FN-BMD (β_IVW_ = 0.144, *P*_IVW_ = 0.028). Most of these causal associations identified by using the IVW method were robust by using other MR methods (Figure 4), demonstrating the stability of the associations. In addition, aspartylphenylalanine was identified to be causally associated with three DXA-BMD (i.e., LS-BMD, FN-BMD, TB-BMD) (Table 1). Levulinate (4-oxovalerate) is significantly associated with fracture risk (OR_IVW_ = 0.843, *P*_IVW_ = 0.032). The scatter and funnel plots, as presented in Supplementary Figures S3, S4, ruled out the existence of potential horizontal pleiotropy for all the identified metabolites.

**Table 1:** Causal metabolites were identified and validated by MR analysis for BMD traits.

**Metabolite**	**eBMD (GRS)**		**eBMD (MR-IVW)**		**LS-BMD (MR-IVW)**		**FN-BMD (MR-IVW)**		**TB-BMD (MR-IVW)**	
	**BETA (95%CI)**	***P***	**BETA (95%CI)**	***P***	**BETA (95%CI)**	***P***	**BETA (95%CI)**	***P***	**BETA (95%CI)**	***P***
ADpSGEGDFXAEGGGVR	−0.021 (−0.027, −0.014)	8.59E-11	−0.207 (−0.353, −0.406)	0.005	−0.369 (−0.534, −0.719)	1.08E-05	/	/	/	/
1-palmitoleoyl glycero phosphocholine	0.036 (0.025, 0.047)	1.12E-10	0.302 (0.089, 0.592)	0.005	/	/	/	/	/	/
Arachidonate (20:4n6)	−0.020 (−0.027, −0.013)	2.85E-08	−0.197 (−0.305, −0.387)	3.39E-04	/	/	/	/	/	/
Phenol sulfate	0.013 (0.009, 0.018)	1.12E-07	0.186 (0.062, 0.365)	0.003	/	/	/	/	0.164 (0.035, 0.558)	0.012
1-linoleoyl glycero phosphoethanolamine	0.018 (0.011, 0.025)	4.48E-07	0.184 (0.063, 0.360)	0.003	/	/	/	/	/	/
Aspartyl phenylalanine	0.016 (0.009, 0.022)	2.73E-06	/	/	0.223 (0.011, 0.645)	0.039	0.241 (0.056, 1.198)	0.011	0.146 (0.011, 0.297)	0.034
Isobutyry-l-carnitine*	0.010 (0.006, 0.014)	3.63E-06	0.117 (0.072, 0.230)	4.08E-07	/	/	0.144 (0.016, 0.852)	0.028	/	/
1-arachidonoyl glycerol phosphocholine	−0.013 (−0.018, −0.007)	2.88E-05	−0.136 (−0.215, −0.266)	8.01E-04	/	/	/	/	/	/

* Represents metabolites reaching the significance level of 0.05 even after Bonferroni adjustment. eBMD, estimated bone mass density; GRS, genetic risk score; MR, Mendelian randomization; IVW, Inverse-variance weighted; CI, Confidence interval; eBMD, estimated bone mineral density; TB-BMD, total body bone mineral density; LS-BMD, lumbar spine bone mineral density; FA-BMD, forearm bone mineral density; FN-BMD, femoral neck bone mineral density.

Notably, three metabolites were associated with three or more osteoporosis traits with MR analysis (Supplementary Table S5), although the associations did not reach the conservative significance level under Bonferroni correction with the GRS method. Specifically, epiandrosterone sulfate is positively associated with eBMD, four DXA-BMDs, and fracture; and rosterone sulfate is positively associated with eBMD, fracture, LS- BMD, FN-BMD, and TB-BMD; and N-acetylornithine is positively associated with eBMD, LS-BMD, and FN-BMD.

The sensitive analysis of the above-identified metabolites showed that these associations were robust and would not be affected by outliers and pleiotropy (Supplementary Table S6). LOOCV analysis ruled out the potential large effect of most IVs (Supplementary Table S7). Additional sensitive analysis based on IVs selected under different thresholds proved robustness of our results in most situations (Supplementary Table S8). Furthermore, reverse MR analysis ruled out the possibility of the causal effect of eBMD on the identified metabolites (Supplementary Table S9).

### Multivariable MR analysis

**Figure 5:**
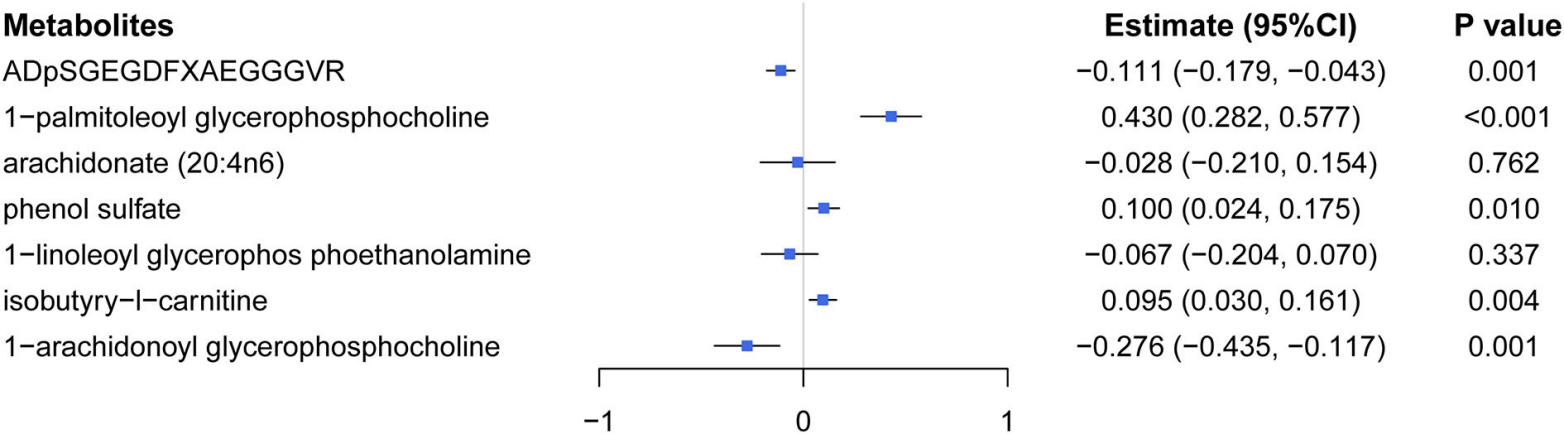
Effects of five significant causal metabolites on eBMD were estimated by using the multivariate MR regression.

Multivariable MR analysis with IVs for all identified metabolites showed that there existed strong evidence of independent causal effects for ADpSGEGDFXAEGGGVR, 1-palmitoleoyl glycerophosphocholine, phenol sulfate, isobutyry-l-carnitine, and 1-arachidonoyl glycerophosphocholine on eBMD (Figure 5), and the directions of adjusted causal effects were consistent with univariable MR analyses. However, the effect of AA and 1-linoleoyl glycerophosphoethanolamine became insignificant in multivariable MR analysis, suggesting potential pleiotropic effects of corresponding IVs. Besides, multivariable MR analysis for DXA-BMD validated the independent causal effect of ADpSGEGDFXAEGGGVR, isobutyry-l-carnitine, and phenol sulfate (Supplementary Table S10).

## Discussion

Integrating large-scale GWAS individual-level data and summary statistics, with an attempt to systematically reveal the underlying association mechanism among blood metabolites and osteoporosis from a genetic perspective, this study generated robust evidence supporting that blood metabolites could causally affect BMDs and fracture risk. Utilizing genetic variants as proxies, 18 metabolites were identified as significant for eBMD and 1 metabolite significant for fracture with the GRS method. Then, 8 metabolites were further validated by two-sample MR in different osteoporosis phenotypes (7 for eBMD, 1 for fracture, 2 for LS-BMD, 2 for TB-BMD, and 2 for FN-BMD). Besides, causal effects of another three metabolites were identified to consistently influence multiple BMD traits. Furthermore, multivariable MR analyses have validated the independent effects of five metabolites on BMD. Metabolic pathway analysis indicated that the above significant metabolites were enriched in pathways of “caffeine metabolism,” “valine, leucine, isoleucine biosynthesis,” “aminoacyl-tRNA biosynthesis,” “alpha linolenic acid,” “linoleic acid metabolism,” and other pathways.

In the present study, small peptides, such as ADpSGEGDFXAEGGGVR, which is derived from the fibrinogen alpha chain, showed an inverse causal effect on BMD. Such findings were consistent with the observation of a significant inverse correlation between fibrinogen and BMD in a previous clinical study (27). Increases in fibrinogen peptide in the context of post-menopausal status in animals promoted inflammation-driven bone resorption by activating osteoclastogenesis and affecting the number and function of osteoclasts by modulating IL-6 levels (28). All the above findings suggested that a higher level of metabolite of fibrinogen exerts a causal effect on osteoporosis risk by enhancing osteoclast activation and promoting bone loss.

Aspartyl phenylalanine is a major product of aspartame after its decomposition by intramolecular cyclization and demethylation, whereas aspartame is a methyl ester of the N-L-α aspartyl-L phenylalanine dipeptide (29). Following routine oral administration of aspartame, osteoarthritis mouse models presented increased bone density and muscle mass, suggesting potential therapeutic effects of aspartame in other disease situations with bone loss (30). A recent study has reported that supramolecular nanoassemblies of salmon calcitonin and aspartame have a good osteoinductive capacity, providing a convenient alternative strategy for osteoporosis therapy (31), and aspartame was confirmed to improve the biocompatibility and pharmacodynamics of salmon calcitonin. In contrast, the use of aspartame was also found to increase the risk of osteoporosis by interacting with cations (such as Fe^2+^, Ca^2+^, Cd^2+^, and Zn^2+^ ions) and excreting them from the body (32). These inconsistent findings merit further investigation.

There are limited studies investigating the correlations of epiandrosterone sulfate and androsterone sulfate with bone metabolism. However, mentioning this marginal association here is noteworthy since dehydroepiandrosterone sulfate is a precursor of sulfated adrenal androgen (e.g., epiandrosterone sulfate, androsterone sulfate). Our findings, together with previous observational research (33, 34), support associations between dehydroepiandrosterone sulfate and bone phenotypes. Kim et al. reported that the reduced adrenal androgen dehydroepiandrosterone-sulfate (DHEA-S) may contribute to lower trabecular bone score (TBS) in patients with subclinical hypercortisolism (SH) (33). Similarly, Ahn et al. nicely used cross-sectional data of a prospective multicenter project and noted that reduced DHEA-S in post-menopausal women and men may contribute to BMD reduction in Asians with SH (34). Consistent with a previous one-sample MR study (9), we found that sulfated adrenal androgens play a causal role in BMD changes. SNP rs474229 in the CYP3A43 gene was found to be a shared IV for these two sulfated adrenal androgens, while CYP3A43 could encode a member of the cytochrome P450 superfamily of enzymes that play a role in catalyzing reactions involved in drug metabolism, as well as the synthesis of cholesterol, steroids, and other lipids. Moreover, it is well-known that androgens play an active role in bone metabolism, promoting the acquisition of bone mass at puberty and contributing to the maintenance of bone mass later. However, whether the elevated levels of these two sulfated adrenal androgens reflect an elevated synthesis of endogenous androgen, thereby indirectly promoting bone metabolism, or whether they can directly affect bone metabolism is unknown yet.

In our MR analysis, AA involved in the metabolism of (alpha) linolenic acid is identified to be negatively associated with all BMD traits except for FA-BMD and positively correlated with fracture risk. Pathway analysis demonstrates that AA is involved in the alpha linolenic acid and linoleic acid metabolism pathways, which are protective of bone health (35). At the same time, a derivative of AA, 1-arachidonoyl glycero phosphocholine is determined to be positively associated with osteoporosis risk in GRS and MR analysis. Many studies found that AA affects bone metabolism through various mechanisms, mainly *via* the OPG/RANKL/NF-κB pathway mediated by PGE2/EP4 to promote osteoclastogenesis as well as by regulating the imbalance of osteogenic and adipogenic differentiation of MSCs (with the increased ability to differentiate into adipocytes). Consistently, the AA level was abnormally elevated in ovariectomized osteoporotic rats, implying that increased biosynthesis of unsaturated fatty acids could lead to osteoporosis (36). Harris et al. found an inverse association between dietary polyunsaturated fatty acid (PUFA) intake and BMD in post-menopausal women receiving hormonal therapy (37). However, several studies contradicted our results, which could be attributed to unavoidable confounders in population studies during the long-term development of osteoporosis (38, 39).

Consistent with our result, a high concentration of N-acetylornithine (NAC) is associated with an increased risk of high bone turnover, which is a major determinant of osteoporosis in late post-menopausal women (40). On one hand, supplementation with NAC can treat OVX-induced osteoporosis in a mouse model of bilateral ovariectomy by simultaneously reducing osteoclast bone resorption, reactive oxygen species (ROS), and DNA damage (41). On the other hand, NAC (ROS scavenger) may inhibit the differentiation and mineralization of osteoblastic cells *via* ROS-dependent signaling pathways (42). During bone remodeling, bone is continuously renewed and mediated through the balance between osteoblastic bone formation and osteoclastic bone resorption, which might be mediated by NAC through the dual roles of ROS in bone homeostasis (43).

Although few studies had focused on the relationship between osteoporosis and the remaining metabolites identified in the present research, there is an inextricable association between them. As we all know, 1-palmitoleoyl-glycerophosphocholine derives from a palmitoleic acid and inhibits RANKL-induced osteoclastogenesis and bone resorption through inhibition of NF-κB and MAPK signaling pathways (44). As a derivative of linoleic acid, 1-linoleoyl glycero phosphoethanolamine is significantly negatively associated with osteoporosis risk. Conjugated linoleic acid has been frequently proved to decrease adipose mass and improve bone health in mice (45). Therefore, further validation by bio functional assays is required, which is beyond the scope of this study.

Metabolic pathways enriched by most identified metabolites (with a nominally significant association with eBMD) in our results were consistent with previous reports, which might play roles in the development of osteoporosis. Notably, the caffeine metabolism pathway (metabolites: paraxanthine, theobromine, and 1-Methyluric acid) was highlighted as potential targets in both pathways analysis (i.e., SMPDB and KEGG), which has previously been reported to play a crucial role in the development of osteoporosis in previous studies (46). Furthermore, fatty acid-related pathways (such as Alpha Linolenic Acid and Linoleic Acid Metabolism and Fatty Acid Biosynthesis), amino acid metabolism (e.g., Arginine and Proline Metabolism), as well as Bile Acid Biosynthesis, were overlapping signals of the results of both enrichment analyses, which may be potential biochemical mechanisms of osteoporosis (36). Besides, metabolites identified for DXA-BMD were proved to be involved in multiple amino acid metabolism pathways and lipid pathways. However, the relationship between aminoacyl-tRNA biosynthesis, as well as some other pathways (such as Biotin Metabolism) and osteoporosis, has not been determined yet and still awaits investigation.

Compared with previous studies, our research identified different features for osteoporosis based on the large-scale eBMD and fracture data sets. The study from the TwinsUK cohort included 6,055 women (9) and found four biomarkers causally associated with BMD. Two of them were confirmed in our analysis (i.e., androsterone sulfate and epiandrosterone sulfate), whereas others did not reach the significance threshold of 0.05 in GRS or MR analysis. The study from Framingham Heart Study recruited around 5,000 subjects for discovery and replication analysis and reported significant metabolites (8), but none of them were significant herein in our analysis. Comparatively speaking, the present research contained a total of ~400,000 samples with genome-wide genotyping scans, which could greatly improve statistical power. Therefore, the present findings should be quite convincing.

In summary, based on the publicly available metabolomics data, the present study supported that the altered levels of metabolites, such as lipid and amino acid, play a role in the progression of osteoporosis as well as in the development of a bone fracture. The causal associations identified at the genetic variant level will provide a metabolomics perspective on screening and identifying significantly altered metabolites that influence the development of osteoporosis, in parallel with mining and identifying specific metabolic pathways that may be targeted for treatment or intervention in osteoporosis. In turn, it is promising to better predict which individuals will suffer an upcoming event of rapid bone loss or even develop osteoporosis, and to help people predisposed to osteoporosis achieve normal bone metabolism through dietary and pharmacologic interventions. However, several limitations exist in our research. First, due to the lack of data, it is infeasible for us to evaluate the relationship between GRS of metabolites on DXA-BMD. Second, the present analysis was conducted on European ancestry, and the findings, probably, cannot be extended to other ethnicities/races. Finally, we could not evaluate metabolite GRSs constructed due to the lack of individual level data.

## Data availability statement

Publicly available datasets were analyzed in this study. This data can be found here: https://www.ukbiobank.ac.uk; http://www.gefos.org/; http://metabolomics.helmholtz-muenchen.de/gwas/. All codes and data sets used in the research are available at https://github.com/biostatYu/MRcode/.

## Ethics statement

The studies involving human participants were reviewed and approved by North West Multi-centre Research Ethics Committee (MREC). The patients/participants provided their written informed consent to participate in this study.

## Author contributions

F-YD, S-FL, R-RC, and X-HY conceived the design of the study. X-HY and LZ obtained the data. R-RC and X-HY cleared up the datasets and mainly performed the data analyses. F-YD, S-FL, R-RC, Y-QY, and X-HY drafted and revised the manuscript. All authors approved the manuscript and provided relevant suggestions.

## Funding

This study was supported by Natural Science Foundation of China (81872681, 82173529, 82173598, and 82103922), the Science and Technology Project of Suzhou (SS202050 and SYS2019024), and the QingLan Project of Higher Education of Jiangsu Province, a Project of the Priority Academic Program Development of Jiangsu Higher Education Institutions and Postgraduate Research & Practice Innovation Program of Jiangsu Province (KYCX22_3227).

## Conflict of interest

The authors declare that the research was conducted in the absence of any commercial or financial relationships that could be construed as a potential conflict of interest.

## Publisher's note

All claims expressed in this article are solely those of the authors and do not necessarily represent those of their affiliated organizations, or those of the publisher, the editors and the reviewers. Any product that may be evaluated in this article, or claim that may be made by its manufacturer, is not guaranteed or endorsed by the publisher.
