# Supplementary material for: Systematic evaluation for the causal effects of blood metabolites on osteoporosis: Genetic risk score and Mendelian randomization
Source: Front Public Health. 2022 Aug 25;10:905178. doi: 10.3389/fpubh.2022.905178 (PMC9452842; doi:10.3389/fpubh.2022.905178)
Supplement: Supplementary file 1 [file Data_Sheet_1.docx]

Supplementary file

# Supplementary tables and figures


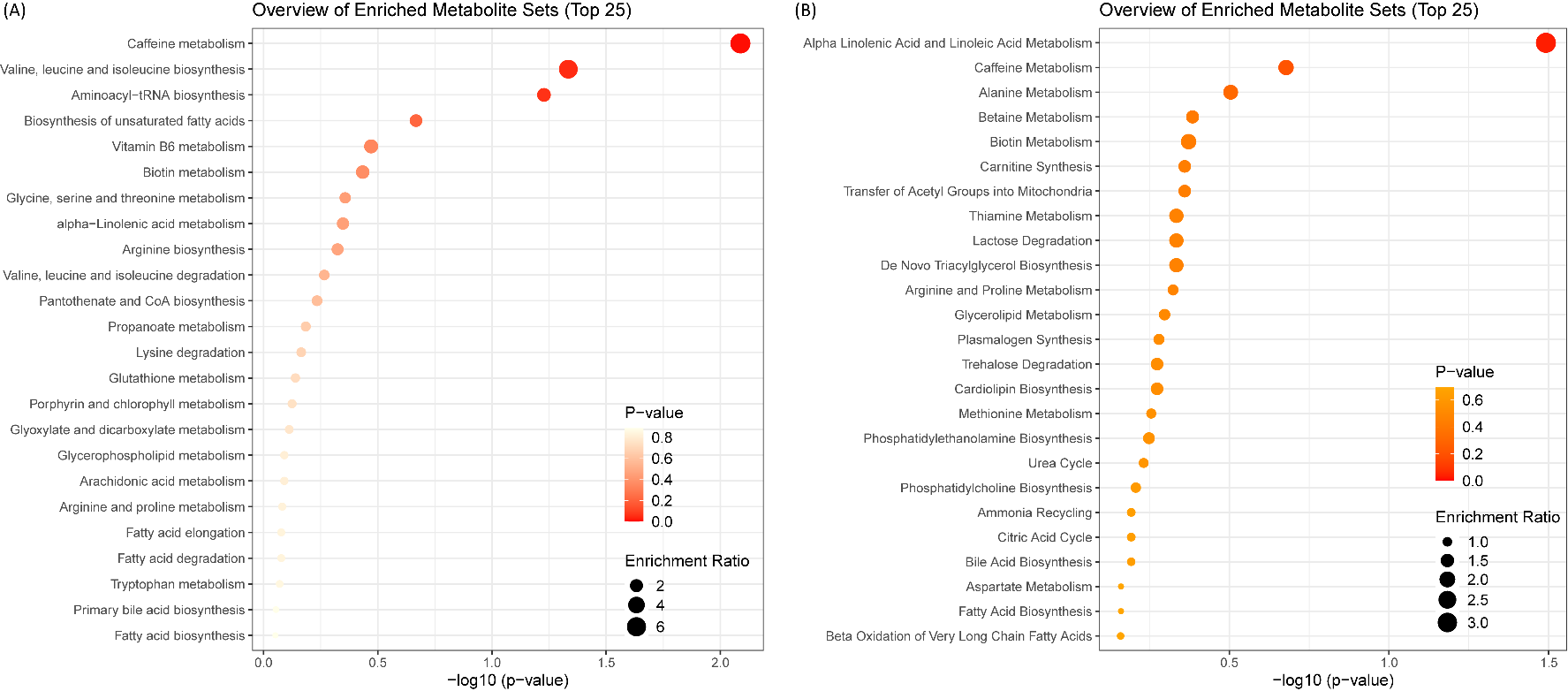


Figure S1. Metabolic pathways analysis with all identified eBMD-related metabolites based on KEGG (A) and SMPDB (B) databases.


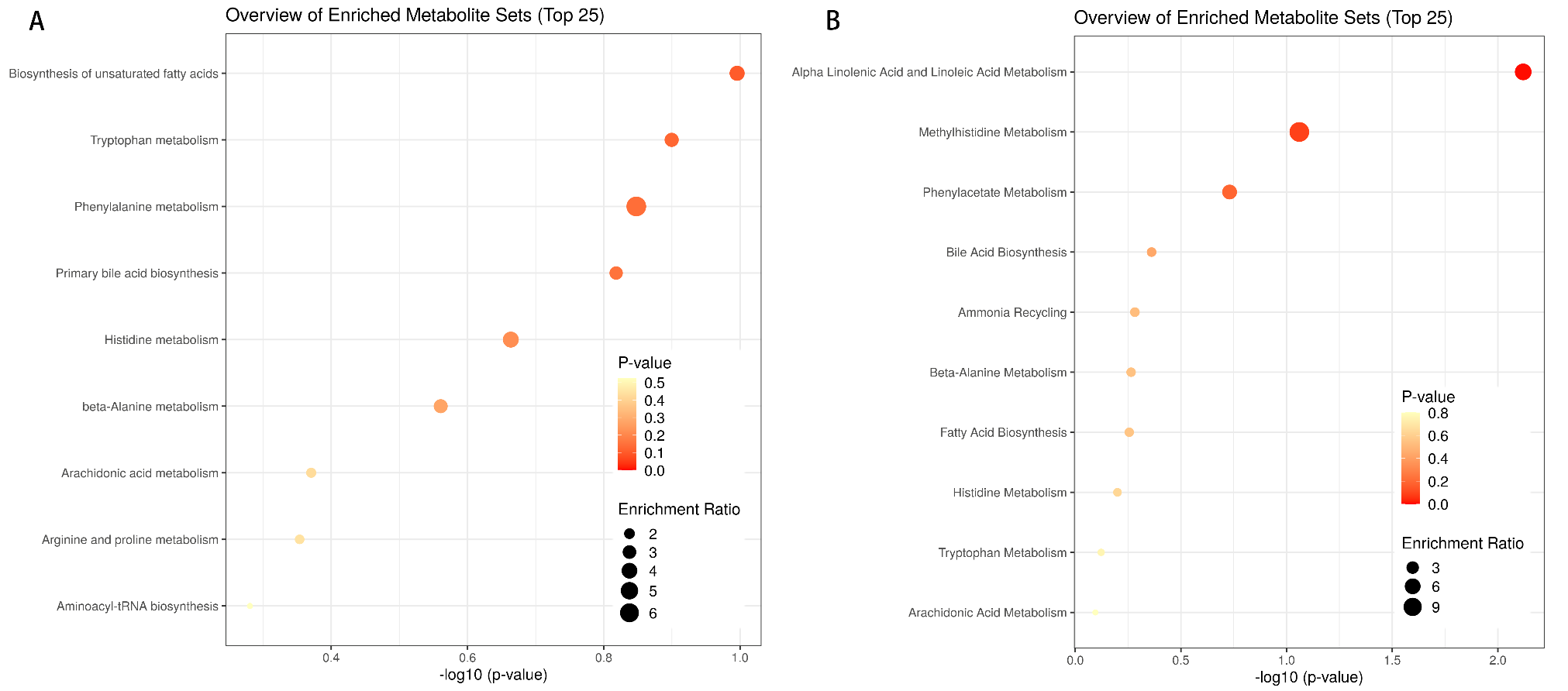


Figure S2. Metabolic pathways analysis with all identified fracture-related metabolites based on KEGG (A) and SMPDB (B) databases.


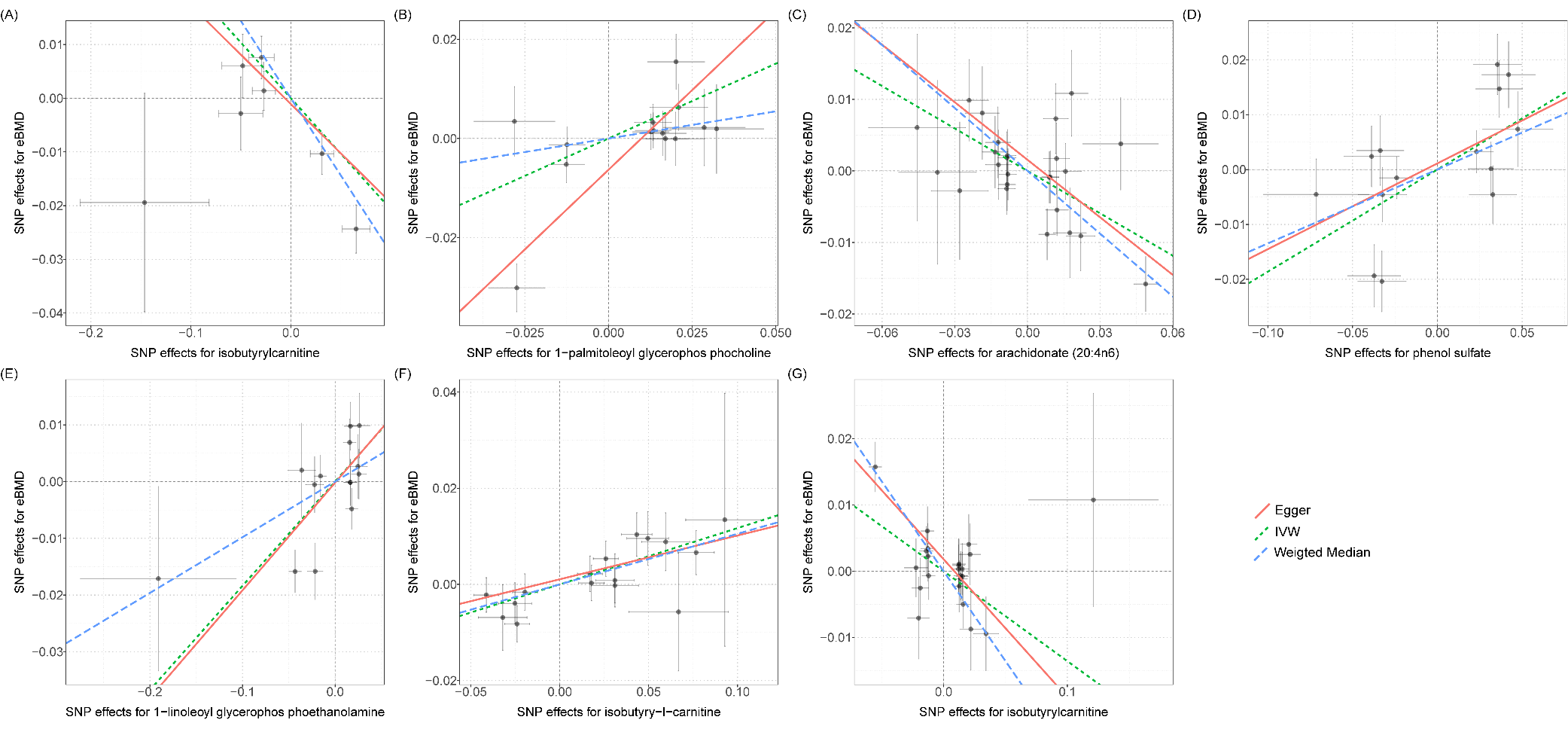


Figure S3. Scatter plot for the relationship between metabolites and eBMD. (**A**) *isobutyrylcarnitine*; (**B**) 1-palmitoleoyl glycerophos phocholine; (**C**) arachidonate (20:4n6); (**D**) phenol sulfate; (**E**) 1-linoleoyl glycerophos phoethanolamine; (**F**) isobutyry-l-carnitine; (**G**) isobutyry-l-carnitine. The red lines represents the MR-Egger estimate, green lines represent the IVW estimate and blue lines represent the weighted median estimate.


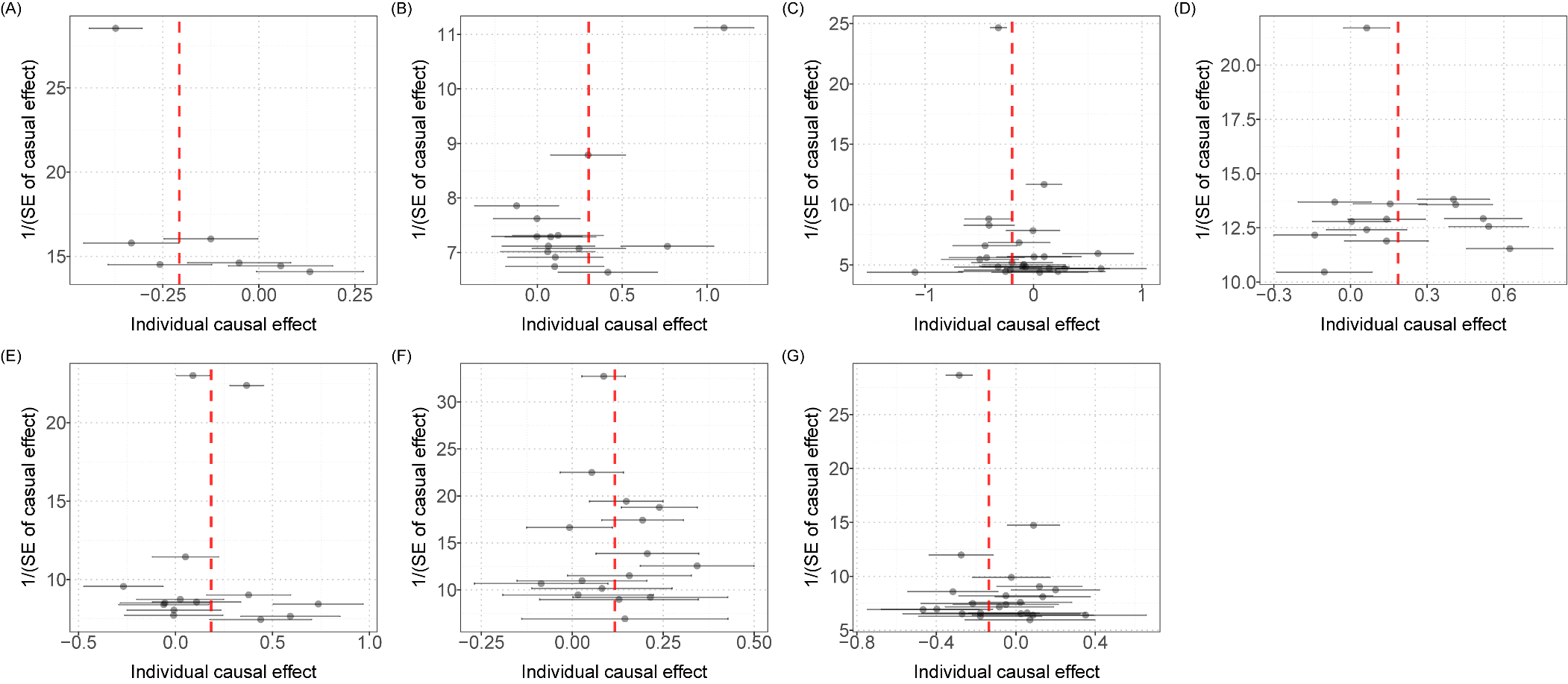


Figure S4. Funnel plot for the relationship between metabolites and eBMD. (**A**) *isobutyrylcarnitine*; (**B**) 1-palmitoleoyl glycerophos phocholine; (**C**) arachidonate (20:4n6); (**D**) phenol sulfate; (**E**) 1-linoleoyl glycerophos phoethanolamine; (**F**) isobutyry-l-carnitine; (**G**) isobutyry-l-carnitine. The red dashed line represents the IVW estimate.

Table S1. Summary information for the genetic data used in the present study.

Table S2. The instrumental variables for significant metabolites used in MR analyses.

Table S3. Causal association of Metabolites on eBMD estimated by both GRS and MR.

Table S4. Functional and integrative analysis of identified metabolites.

Table S5. Causal effect of three additional metabolites identified in MR analysis.

Table S6. MR analysis of all metabolites identified in MR analysis.

Table S7. LOOCV analysis of identified metabolites on outcomes

Table S8. Causal effects of BMD on five identified metabolites.

Table S9. Multivariable MR analysis for DXA-BMD.
